# Supplementary material for: Improvements in Quality of Life and Thyroid Parameters in Hypothyroid Patients on Ethanol-Free Formula of Liquid Levothyroxine Therapy in Comparison to Tablet LT4 Form: An Observational Study
Source: J Clin Med. 2021 Nov 10;10(22):5233. doi: 10.3390/jcm10225233 (PMC8624226; doi:10.3390/jcm10225233)
Supplement: Supplementary file 1 [file jcm-10-05233-s001.zip › jcm-1424356-supplementary.pdf]

Journal of
Clinical Medicine

MDPI

SUPPLEMENTARY MATERIAL

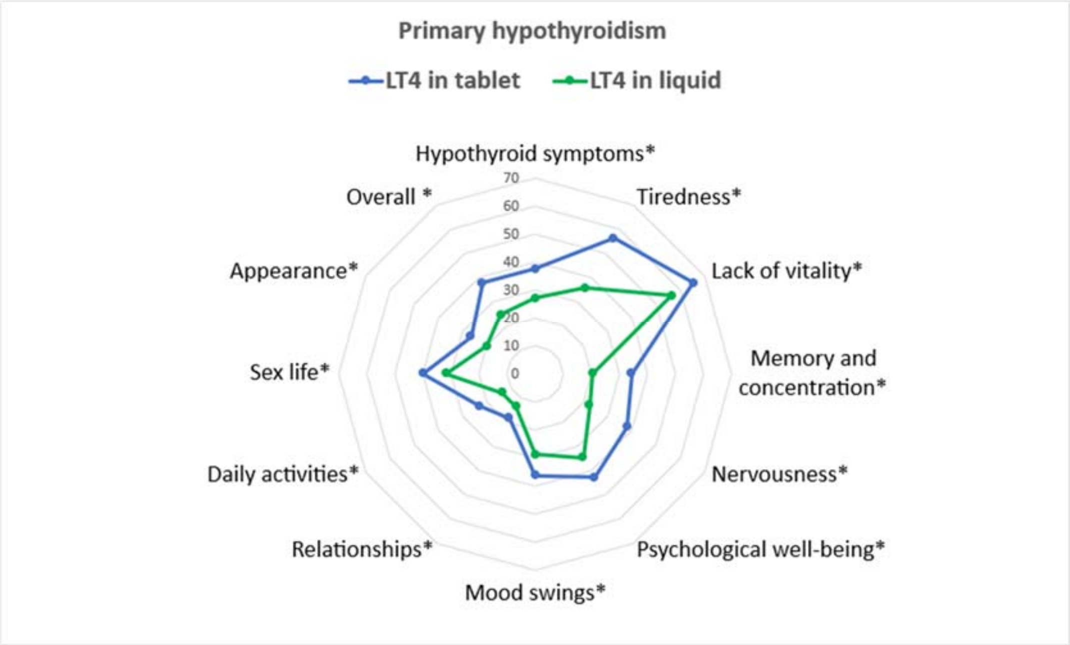

(a) *, P<0.05.

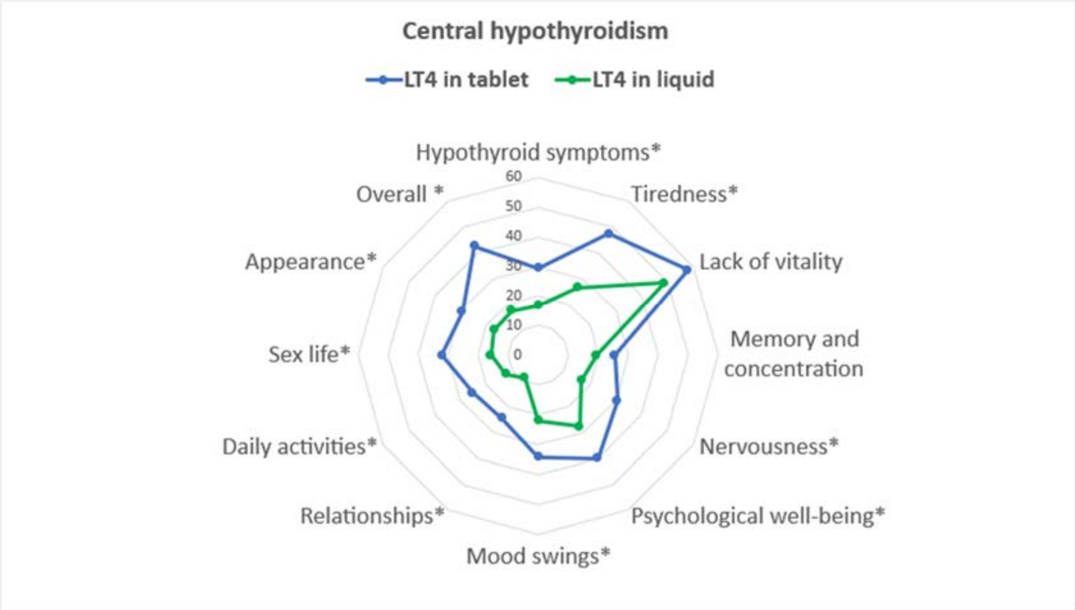

(b) *, P<0.05.

Figure S1. Spider plots based on data summarized in table 2 for better visualization: changes in QoL in patients with PH (a) and CH (b) after 8 weeks of liquid LT4 therapy. The indicated area is inversely proportional to the QoL (the smaller area the higher QoL).
